# Cardiovascular safety of propranolol oral solution in infantile hemangiomas: a disproportionality analysis based on the FAERS database

**DOI:** 10.1038/s41598-025-07577-3

**Published:** 2025-07-07

**Authors:** Wenjia Nie, Liyun Zheng, Yuting Xia, Dongping Xiao, Yun Zou

**Affiliations:** 1 Dermatology Hospital of Jiangxi Province, Jiangxi Provincial Clinical Research Center for Skin Diseases, Candidate Branch of National Clinical Research Center for Skin Diseases, JXHC Key Laboratory of Skin Infection and Immunity, The Affiliated Dermatology Hospital of Nanchang University, Nanchang, 330000 Jiangxi China; 2 Department of Psychiatry, Jiangxi Medical College, Jiangxi Mental Hospital & Affiliated Mental Hospital, Nanchang University, Nanchang, 330029 Jiangxi China; 3 Nanchang City Key Laboratory of Biological Psychiatry, Jiangxi Provincial Clinical Research Center on Mental Disorders, Jiangxi Mental Hospital, Nanchang, 330029 Jiangxi China; 4 Department of Dermatology, Union Hospital, Tongji Medical College, Huazhong University of Science and Technology, Wuhan, 430014 Hubei China; 5 Department of Cardiology, The First Hospital of Nanchang, Nanchang, 330000 Jiangxi China; 6 Department of Plastic Surgery, Jiangxi Provincial Children’s Hospital, Nanchang, 330000 Jiangxi China

**Keywords:** Propranolol oral solution, Cardiovascular safety, FAERS database, Real-world study, Infantile hemangiomas, Drug safety, Skin diseases, Skin manifestations, Paediatric research

## Abstract

This study aims to explore the potential association between propranolol and cardiovascular adverse events (AEs) through real-world evidence. Reports from the FDA Adverse Event Reporting System (FAERS) database spanning from January 2014 to September 2024, identifying propranolol oral solution as the primary suspected (PS) drug, were analyzed. Disproportionality analysis employed four key metrics: Reporting Odds Ratio (ROR), Proportional Reporting Ratio (PRR), Bayesian Confidence Propagation Neural Network (BCPNN), and Multi-item Gamma-Poisson Shrinkage (MGPS) to identify positive signals of cardiovascular AEs potentially associated with propranolol oral solution. Among 7,518 AE reports where propranolol oral solution was the PS drug, 196 cardiovascular AEs were identified. Peripheral coldness (*n* = 131, ROR 85.58, PRR 84.11), cyanosis (*n* = 24, ROR 15.91, PRR 15.87), and pallor (*n* = 12, ROR 3.79, PRR 3.79) were potentially associated with propranolol oral solution use. However, no positive signals were observed for other cardiovascular AEs, such as hypotension, bradycardia, or arrhythmias. Apart from peripheral vascular changes, our analysis did not detect positive signals for severe cardiovascular AEs, suggesting a favorable cardiovascular safety profile, though FAERS limitations such as underreporting warrant cautious interpretation. To ensure safety, standardized use and monitoring remain crucial.

## Introduction

Since the discovery of propranolol as a treatment for infantile hemangiomas in 2008, studies have confirmed its efficacy^1,2^. In 2014, propranolol oral solution became the first drug approved by the FDA for the treatment of infantile hemangiomas^3^. However, its systemic safety with oral administration, particularly regarding cardiovascular effects, remains a concern for physicians and families^4^.

As a vulnerable population, infants and young children may be more susceptible to severe cardiovascular adverse events when using propranolol, including hypotension, bradycardia, and conduction block^5^. Although two recent retrospective cohort studies have demonstrated a favorable safety profile for propranolol oral solution, including a low incidence of cardiovascular AEs^6,7^, its cardiovascular safety has not been fully investigated due to limitations such as small sample sizes and selection bias.

The FDA Adverse Event Reporting System (FAERS) database, a large-scale real-world pharmacovigilance dataset, offers a valuable resource for such investigations^8^. It contains AE reports submitted by healthcare professionals and consumers since the market approval of propranolol oral solution. Disproportionality analysis is particularly suitable for detecting potential associations between propranolol oral solution and cardiovascular AEs^9^. A positive signal suggests a suspected relationship and may indicate a possible safety concern^10^.

To improve understanding of the cardiovascular safety profile of propranolol oral solution and support safer clinical use, we conducted a retrospective pharmacovigilance study using the FDA Adverse Event Reporting System. By applying multiple disproportionality analysis methods, we aimed to identify potential cardiovascular safety signals—particularly for serious events such as bradycardia, hypotension, and atrioventricular block.

## Method

### Data sources

Following the market approval of propranolol oral solution, this study analyzed adverse event (AE) reports from the FAERS database, covering data from Q1 2014 to Q3 2024. Data were extracted from the FAERS public dashboard (https://fis.fda.gov/extensions/FPD-QDE-FAERS/FPD-QDE-FAERS.html) on December 2024, and R software (version 4.3.3) was used to import, process, and conduct disproportionality analyses.

### Data extraction and analysis

The FAERS database consists of seven core datasets—patient demographics (DEMO), drug information (DRUG), adverse events (REAC), outcomes (OUTC), report sources (RPSR), therapy dates (THER), and indications (INDI)—as well as a Deleted file containing withdrawn or merged cases. For records sharing the same case ID in the DEMO table, the report with the latest submission date was retained to complete the deduplication process. Datasets were then merged using the primary ID to retrieve matched records from DRUG, REAC, and other related tables. Cases involving propranolol oral solution were identified by searching its trade names, HEMANGEOL and HEMANGIOL. Reports listing propranolol oral solution as the primary suspected (PS) drug were extracted, including the reporting date, patient demographics (age and gender), reporter type, and geographic location.

This study employed multiple disproportionality analysis methods to identify AE signals, including the reporting odds ratio (ROR)^11^, proportional reporting ratios (PRR)^12^, Bayesian Confidence Propagation Neural Network (BCPNN)^13^, and Multi-item Gamma Poisson Shrinker (MGPS)^14^. Each method has certain limitations: ROR may yield unstable estimates when event counts are low, while PRR may fail to detect true signals in datasets with sparse reporting. BCPNN is sensitive to prior assumptions and may underestimate signal strength in heterogeneous datasets. MGPS may overly shrink frequent events, potentially masking true associations. These limitations contribute to the combined application of all four methods, enhancing detection sensitivity and reducing the risk of missing potential signals^15^.

All methods were based on 2 × 2 contingency tables, with relevant formulas and thresholds summarized in Table 1. These thresholds have been widely adopted in previous signal detection studies to balance sensitivity and specificity, and to ensure statistical robustness when the number of reports (a) is at least three. Positive signals exceeding the threshold may indicate a potential association requiring further evaluation. Conversely, the absence of signals meeting predefined thresholds may reflect a low reporting frequency of the evaluated events and may be interpreted as supportive of the drug’s safety in this context.

**Table 1:** ROR, PRR, BCPNN, and EBGM calculation formulas and their thresholds.

Algorithms	Calculation formulas	Threshold
ROR	(1) ROR = (a/b)/(c/d) = ad/bc (2) 95%CI = e^ln(ROR)±1.96(1/a+1/b+1/c+1/d)^0.5^	95%CI (lower limit) > 1, a ≥ 3.
PRR	(1) PRR = [a(c + d)]/[c(a + b)] (2) ² = [(ad-bc)^2](a + b + c + d)/[(a + b)(c + d)(a + c)(b + d)]	PRR ≥ 2, ² ≥ 4, a ≥ 3.
BCPNN	(1) IC = log_2_[a(a + b + c + d)/(a + b)/(a + c)] (2) 95%CI = E(IC) ± 2 V(IC)^0.5	IC_025_ > 0.
MGPS	(1) EBGM = a(a + b + c + d)/(a + c)/(a + b) (2) 95%CI = e^ln(EBGM)±1.96(1/a+1/b+1/c+1/d)^0.5^	EBGM_05_ > 2.

a: The number of reports that include both propranolol oral solution and the target adverse event (AE). b: The number of reports that include propranolol oral solution with AEs other than the target AE. c: The number of reports that include the target AE with drugs other than propranolol oral solution. d: The number of reports that include both other drugs and AEs other than the target AE. *CI* confidence interval, *IC* information component, *IC025* the lower limit of 95% CI of the IC, *E(IC)* the IC expectations, *V(IC)* the variance of IC, *EBGM* empirical Bayesian geometric mean, *EBGM05* the lower limit of 95%CI of EBGM.

AEs in the FAERS database are coded using Preferred Terms (PTs) from the Medical Dictionary for Regulatory Activities (MedDRA), which classifies these terms into 27 System Organ Classes (SOCs). Signal strength for AEs associated with propranolol oral solution was assessed at the PT level. Given that disproportionality analyses may produce spurious signals when based on very small counts, only PTs with at least three reports were included in the analysis across all four algorithms. Additionally, to improve the clinical interpretability of the findings, PTs with vague or nonspecific descriptions (e.g., “ILLNESS,” “CONDITION AGGRAVATED”) and those lacking biological plausibility (e.g., “HAEMANGIOMA,” “COVID-19”) were excluded.

## Results

### Basic characteristics of reported AEs

Between Q1 2014 and Q3 2024, a total of 7,518 reports involving 3,277 patients identified propranolol as the PS drug associated with adverse events. Detailed characteristics of these adverse events are presented in Table 2.

**Table 2:** Characteristics on adverse events among propranolol oral solution users from the FAERS database.

Characteristics	Adverse events among propranolol oral solution users (*N* = 3277)
Gender, *n* (%)	
Female	2057 (62.8%)
Male	807 (24.6%)
Missing data	413 (12.6%)
Age (years), n (%)	
< 1	445 (13.6%)
≥ 1	101 (3.1%)
Missing data	2731 (83.3%)
Weight, n (%)	
<4.5 kg	201 (6.1%)
4.5～8.5 kg	1480 (45.2%)
8.5 ~ 12.5 kg	749 (22.9%)
≥ 12.5 kg	191 (5.8%)
Missing data	656 (20.0%)
Reporters, n (%)	
Health professional	2183 (66.6%)
Consumer	835 (25.5%)
Others	259 (7.9%)
Reported countries, n (%)	
United States	3167 (96.6%)
Non-US	110 (3.4%)
Reporting year, n (%)	
2014 ~ 2023	1505 (45.9%)
2024 (Q1-Q3) *	1772 (54.1%)
Outcome, n (%)	
Overall cases	3014 (92.0%)
Death	7 (0.2%)
Disability	1 (0.0%)
Hospitalization	121 (3.7%)
Life-threatening	9 (0.3%)
Other serious outcomes	125 (3.8%)
Non-serious outcomes	2751 (83.9%)
Days adverse events occurred, n (%)	
0–30 days	102 (3.1%)
31–60 days	21 (0.6%)
61–90 days	20 (0.6%)
91–120 days	14 (0.4%)
121–150 days	19 (0.6%)
> 150 days	45 (1.3%)
Missing data	3007 (94.3%)

*The first, second and third quarter of 2024. *AEs* adverse events, *n* number of cases.

### Cardiovascular signal detection of propranolol oral solution at the PT level

**Fig. 1:**
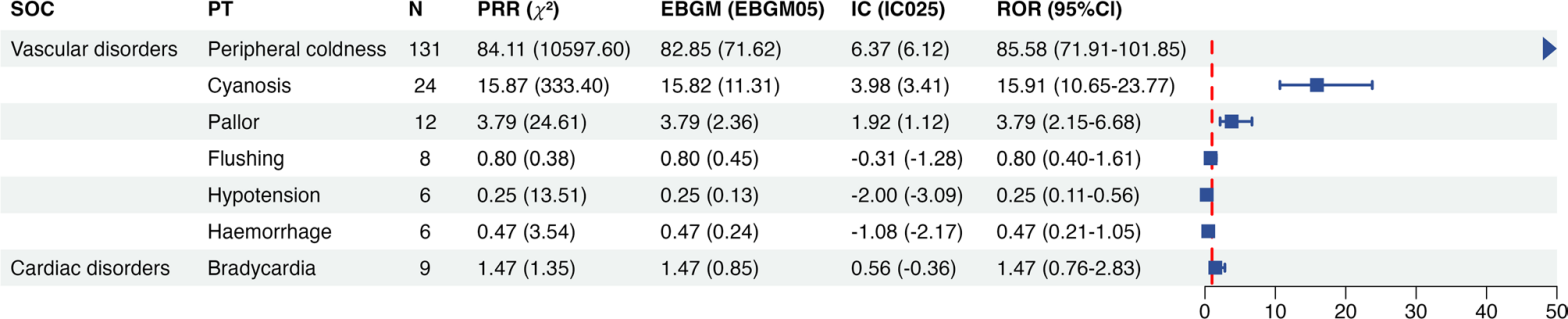
The signal strength of cardiovascular adverse events of propranolol oral solution at the preferred term (PT) level in the FAERS database. *SOC* System organ classes, *ROR* Reporting odds ratio, *PRR* Proportional reporting ratio, *BCPNN* Bayesian confidence propagation neural network, *MGPS* Multi-item gamma poisson shrinker.

Signal detection was performed for all PTs under the SOCs of Vascular Disorders (SOC code: 10047065) and Cardiac Disorders (SOC code: 10007541). No positive signals were identified for severe cardiovascular adverse events, such as bradycardia, hypotension, or atrioventricular block (Fig. 1). Only three PTs demonstrated positive signals: peripheral coldness (*n* = 131, ROR 85.58, PRR 84.11, IC 6.37, EBGM 82.85), cyanosis (*n* = 24, ROR 15.91, PRR 15.87, IC 3.98, EBGM 15.82), and pallor (*n* = 12, ROR 3.79, PRR 3.79, IC 1.92, EBGM 3.79) (Fig. 1).

### Non-cardiovascular signal detection of propranolol oral solution at the PT level

**Fig. 2:**
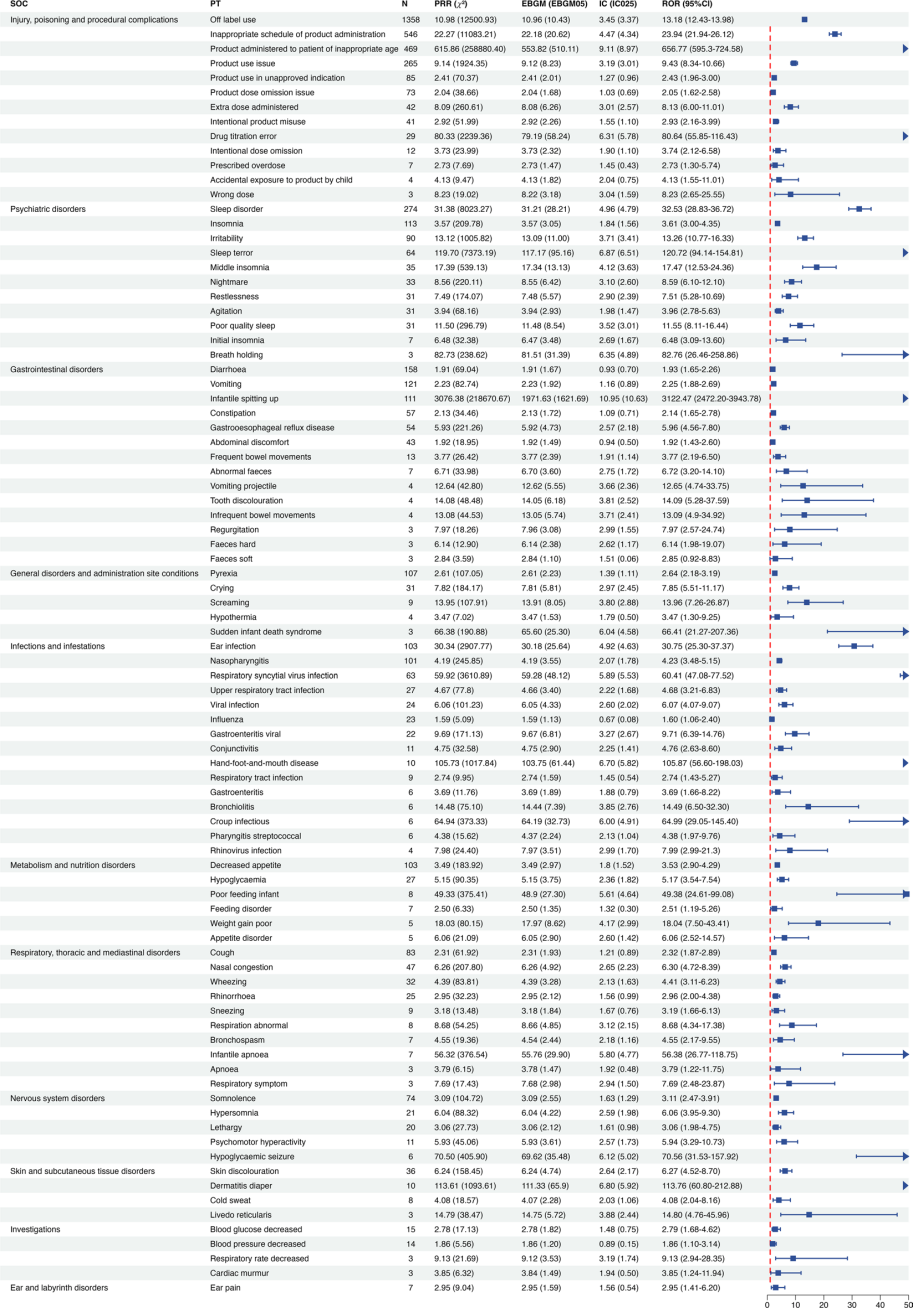
The signal strength of non-cardiovascular adverse events of propranolol oral solution at the preferred term (PT) level in FAERS database. *SOC* System organ classes, *ROR* Reporting odds ratio, *PRR* Proportional reporting ratio, *BCPNN* Bayesian confidence propagation neural network, *MGPS* Multi-item gamma poisson shrinker.

To assess the reliability of the cardiovascular signal findings, the scope of signal detection was expanded to include all PTs across non-cardiovascular SOCs, aiming to comprehensively evaluate the adverse event profile of propranolol oral solution. A total of 88 PTs demonstrated positive signals, as detected by at least one algorithm (Fig. 2). These PTs were distributed across 11 SOC categories, primarily related to drug misuse, neuropsychiatric symptoms, gastrointestinal disturbances, infections, respiratory conditions, and metabolic or nutritional disorders.

## Discussion

Although cardiovascular AEs have been reported in the FAERS database, our analysis did not detect any positive signals indicating an association between propranolol use and severe cardiovascular events, apart from certain peripheral vascular manifestations. In addition, adverse events associated with propranolol oral solution primarily involve the gastrointestinal, respiratory, and neuropsychiatric systems, with commonly reported concerns including decreased appetite, cold extremities, and hypoglycemia, which are generally consistent with the known pharmacological profile of propranolol^16^.

In our analysis, propranolol oral solution was linked to common adverse events and potential risks such as hypoglycemia and bronchial asthma, but no evidence of a link with severe cardiovascular events was observed^4,17^. Moreover, previous studies have shown that while higher doses of propranolol (3 mg/kg/day) are associated with increased rates of adverse events compared to moderate doses (2 mg/kg/day), the incidence of hypotension and bradycardia remains low^18^. Taken together, the available evidence does not point to major cardiovascular safety concerns within the therapeutic dose range.

In addition, the pediatric-specific oral solution may help reduce the cardiovascular risks associated with propranolol. Unlike extemporaneously prepared solutions from adult tablets or capsules, this formulation is specifically designed for infants and young children^19^. Studies suggest that the oral solution may offer improved safety over tablet forms, potentially due to more precise dose adjustment and consistent gastrointestinal absorption^6,7^. This may indicate a more favorable cardiovascular safety profile for the oral solution compared to modified adult tablet preparations in pediatric populations.

Among the reported cardiovascular AEs, peripheral symptoms such as cold extremities, cyanosis, and pallor appeared more frequently in association with propranolol use. Although these peripheral symptoms are described in the drug label as possible manifestations or early indicators of serious cardiovascular AEs^3^, current evidence does not appear to support a direct link. If a strong relationship existed, one would expect to observe higher reporting frequencies of related events, such as hypotension, bradycardia, or atrioventricular block. Moreover, previous studies have reported no occurrences of symptomatic hypotension or bradycardia during the monitoring of propranolol treatment for infantile hemangiomas^5,20^. These findings suggest that such symptoms may not imply serious cardiovascular toxicity but instead reflect the peripheral vascular effects of propranolol^21^.

Although no positive signals were detected for serious cardiovascular AEs in this study, several limitations should be acknowledged. First, the large number of reports classified as “injury, poisoning, and procedural complications” (*n* = 3,085, 94.14%) may lower the relative proportion of cardiovascular AEs, thereby reducing the sensitivity of signal detection for low-frequency cardiovascular events. Second, as a spontaneous reporting system, the FAERS database is subject to inherent biases, including underreporting of adverse events. To assess the impact of this bias, we extended signal detection to non-cardiovascular AEs. Although the results suggest a limited influence, such bias cannot be entirely excluded. Furthermore, although this study used disproportionality analysis rather than absolute frequencies to mitigate the impact of duplicate reporting, if duplication is disproportionately concentrated in non-cardiovascular events, it may still reduce the relative signal strength of cardiovascular AEs.

Additionally, this study has limitations related to demographic completeness and sample composition, which may affect the generalizability of its findings. The age data were missing in 83.3% of cases, limiting the ability to accurately identify the target population of infants and young children, even though infantile hemangioma is the sole approved indication for propranolol oral solution. In addition, the absence of age and weight data limited our ability to assess the inclusion of developmentally vulnerable infants, which may affect the generalizability of the findings to high-risk pediatric populations. Furthermore, because FAERS primarily includes reports from U.S. patients, only 3.4% originated from outside the United States, which may limit the applicability of the findings to other regions or populations. Given these limitations, the results should be interpreted with caution and considered hypothesis-generating rather than confirmatory.

Considering these limitations, ensuring the safe use of propranolol oral solution may still require standardized administration protocols and appropriate monitoring. Clinicians are advised to perform baseline cardiovascular assessments, educate caregivers^22,23^, and remain vigilant during high-risk situations—such as infections, feeding difficulties, or low body weight—where temporary treatment interruption or dose adjustment may help prevent severe adverse outcomes^24,25^. Pediatric-specific formulations should be preferred over modified adult tablets to ensure dosing accuracy and consistent gastrointestinal absorption. Meanwhile, further studies using prospective designs or electronic health records are warranted to validate these findings, particularly in high-risk subgroups such as preterm or low birth weight infants.

## Conclusion

This study, based on real-world data from the FAERS database, did not detect any positive signals of severe cardiovascular AEs associated with propranolol oral solution, which may reflect a favorable cardiovascular safety profile. However, given the inherent limitations of the FAERS database, the absence of positive signals should not be interpreted as evidence of the complete absence of cardiovascular risk. To further promote safe use in clinical practice, standardized administration protocols and appropriate monitoring remain essential.

## Data Availability

Data supporting the results of this study were obtained from the publicly accessible FAERS database (https://fis.fda.gov/extensions/FPD-QDE-FAERS/FPD-QDE-FAERS.html).
